# Supplementary material for: Probabilistic harmonization and annotation of single‐cell transcriptomics data with deep generative models
Source: Mol Syst Biol. 2021 Jan 25;17(1):e9620. doi: 10.15252/msb.20209620 (PMC7829634; doi:10.15252/msb.20209620)
Supplement: Supplementary file 2 — Appendix [file MSB-17-e9620-s001.pdf]

Probabilistic Harmonization and Annotation of Single-cell Transcriptomics Data with Deep Generative Models

Chenling Xu*[1], Romain Lopez*[2], Edouard Mehlman*[2,3], Jeffrey Regier[4], Michael I. Jordan[2,5] & Nir Yosef†[,1,2,6,7]

[1]Center for Computational Biology
University of California, Berkeley

[2]Department of Electrical Engineering and Computer Sciences
University of California, Berkeley

[3]Centre de Mathématiques Appliquées
Ecole polytechnique, Palaiseau

[4]Department of Statistics
University of Michigan, Ann Arbor

[5]Department of Statistics
University of California, Berkeley

[6]Ragon Institute of MGH, MIT and Harvard

[7]Chan-Zuckerberg Biohub Investigator

* These authors contributed equally to this work.

† Corresponding author. Email: niryosef@berkeley.edu (N.Y.).

References

[Kouw and Loog, 2019] Kouw, W. M. and Loog, M. (2019). A review of domain adaptation without target labels. *IEEE Trans Pattern Analysis Mach Intelligence.*

[Saunders et al., 2018] Saunders, A., Macosko, E. Z., Wysoker, A., Goldman, M., Krienen, F. M., de Rivera, H., Bien, E., Baum, M., Bortolin, L., Wang, S., et al. (2018). Molecular diversity and specializations among the cells of the adult mouse brain. *Cell*, 174(4):1015–1030.

[Welch et al., 2019] Welch, J. D., Kozareva, V., Ferreira, A., Vanderburg, C., Martin, C., and Macosko, E. Z. (2019). Single-cell multi-omic integration compares and contrasts features of brain cell identity. *Cell*, 177(7):1873–1887.

Probabilistic Harmonization and Annotation of Single-cell Transcriptomics data with Deep Generative Models

Supplementary Information

Chenling Xu*[1], Romain Lopez*[2], Edouard Mehlman*[2,3],
Jeffrey Regier[4], Michael I. Jordan[2,5] & Nir Yosef†[,1,2,6,7]

[1]Center for Computational Biology
University of California, Berkeley

[2]Department of Electrical Engineering and Computer Sciences
University of California, Berkeley

[3]Centre de Mathématiques Appliquées
Ecole polytechnique, Palaiseau

[4]Department of Statistics
University of Michigan, Ann Arbor

[5]Department of Statistics
University of California, Berkeley

[6]Ragon Institute of MGH, MIT and Harvard

[7]Chan-Zuckerberg Biohub Investigator

* These authors contributed equally to this work.

† Corresponding author. Email: niryosef@berkeley.edu (N.Y.).

List of Figures

Appendix Figure 1 Schematic of the data harmonization problem. 3
Appendix Figure 2 Robustness analysis for harmonization of the pair of datasets MarrowMT-10x / MarrowMT-ss2 with scVI 3
Appendix Figure 3 Visualization of the output of MAGAN on the DentateGyrus pair of datasets. 4
Appendix Figure 4 Visualization of the benchmark PBMC-8K / PBMC-CITE. . 5
Appendix Figure 5 Visualization of the benchmark MarrowMT 10x / ss2. 6
Appendix Figure 6 Visualization of the benchmark Pancreas InDrop / CEL-Seq2 6
Appendix Figure 7 Visualization of the benchmark DentateGyrus10X - Fluidigm C1 7

Appendix Figure 8	Supplementary study of harmonizing datasets with different cellular composition	8
Appendix Figure 9	Follow-up analysis on continuous trajectory harmonization with scANVI	9
Appendix Figure 10	Evaluation of harmonization metric when data quality is corrupted	9
Appendix Figure 11	Evaluation of harmonization metrics in cross-species comparison	10
Appendix Figure 12	Large-scale data integration with scVI	10
Appendix Figure 13	Annotation results for all four dataset pairs (boxplot)	11
Appendix Figure 14	Annotation results for all four dataset pairs (bubbleplot)	12
Appendix Figure 15	Supplementary study of labels concordance	13
Appendix Figure 16	Other methods of classifying T-cell subsets of the PBMC-Pure dataset	14
Appendix Figure 17	Continuous trajectory simulated using SymSim	15
Appendix Figure 18	Presentation of the simulated dataset used for differential expression benchmarking	16
Appendix Figure 19	The effect of the choice of number of classes on the scANVI model	16
Appendix Figure 20	Performance of scVI and scANVI with a negative binomial (NB) distribution	17
Appendix Figure 21	Differential Expression with a negative binomial version of scVI	18

List of Tables

Appendix Table 1	List of datasets used in this paper.	19
Appendix Table 2	Additional metric for retainment of structure via k-means clusters preservation.	20
Appendix Table 3	Composition of cell-types in the PBMC-8K and the PBMC-CITE dataset	20
Appendix Table 4	Runtime Comparative Analysis.	21
Appendix Table 5	Cell types present in the PBMC-sorted dataset.	21

List of Appendix Notes

A Related work **21**

A.1 Related machine learning litterature . . . 21

A.1.1 Domain adaptation . . . 21

A.1.2 Semi-supervised learning with variational autoencoders . . . 22

A.2 Related scRNA-seq harmonization work . . . 22

A.2.1 Why is harmonization a subtle problem? . . . 22

A.2.2 State-of-the-art approaches . . . 23

A.2.3 The approach taken by this manuscript . . . 23

B Training Information **24**

C Alternative model choices **24**

C.1 Zero-inflation in the context of harmonization . . . 24

C.2 Library-size prior for scVI and scANVI . . . 25

D Hierarchical classification **25**

E Evidence Lower Bound decomposition **26**

Appendix Figures

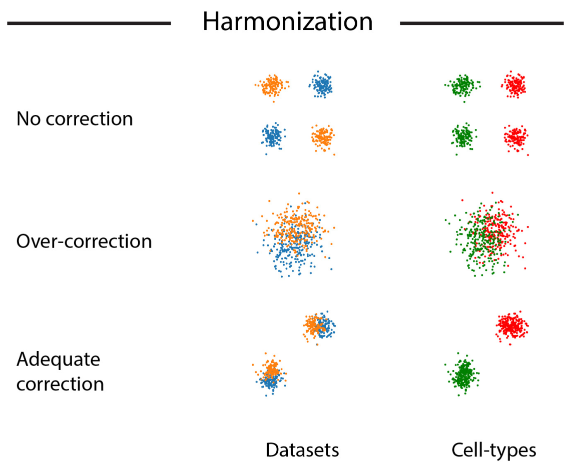

Appendix Figure 1: Schematic of the data harmonization problem. We are provided with two datasets (orange and blue), each consisting of two cell types (red and green). Our evaluation for the harmonization problem consists of two objectives: (1) mixing the two datasets well and (2) retaining the original structure in each dataset. Scenario 1 (top) is the case of under correction where objective (2) is achieved while objective (1) is not. Scenario 2 (middle) is the case of over correction where objective (1) is improved while objective (2) becomes worse. The bottom panel shows the desired scenario of mixing the datasets well while retaining the biological signal.

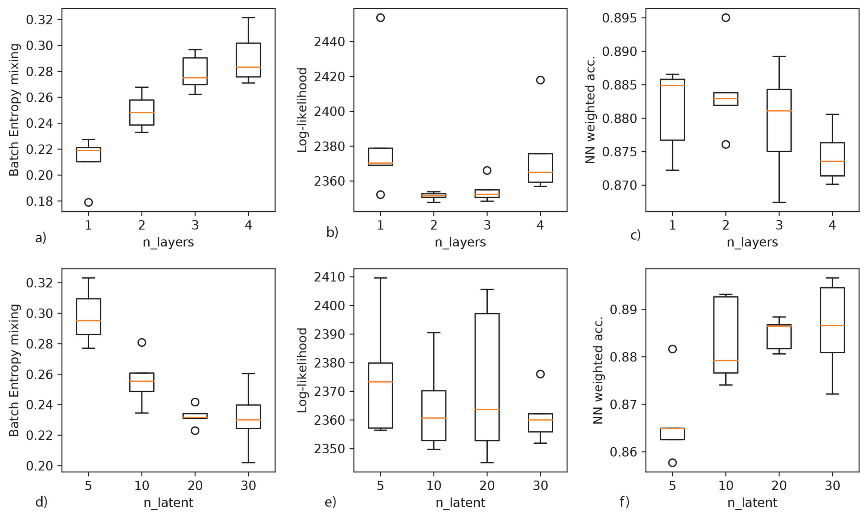

Appendix Figure 2: Robustness analysis for harmonization of the pair of datasets MarrowMT-10x / MarrowMT-ss2 with scVI. $(a-c)$ We augment the number of hidden layers in the neural network f_w and track across $n = 5$ random initializations for the batch entropy mixing (a), the held-out log likelihood (b) and the weighted accuracy of a nearest neighbor classifier on the latent space (c). $(d-f)$ We increase the number of dimensions for the latent variable z and track across $n = 5$ random initialization the batch entropy mixing (d), the held-out log likelihood (e) and the weighted accuracy of a nearest neighbor classifier on the latent space (f).

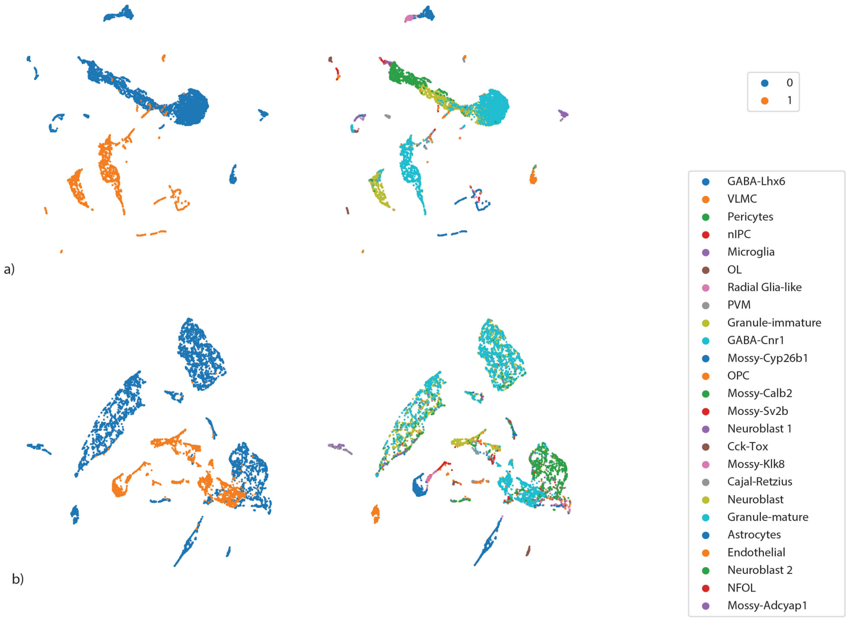

Appendix Figure 3: Visualization of the output of MAGAN on the DentateGyrus pair of datasets. Using MAGAN, we projected the first dataset into the second one (a) and vice-versa (b).

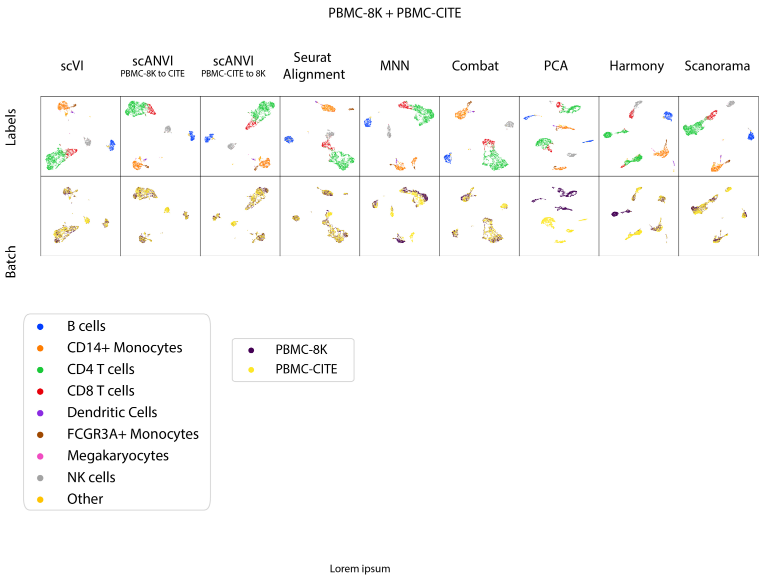

Appendix Figure 4: Visualization of the benchmark PBMC-8K / PBMC-CITE. All positions for the scatter plots are derived using UMAP on the latent space of interest.

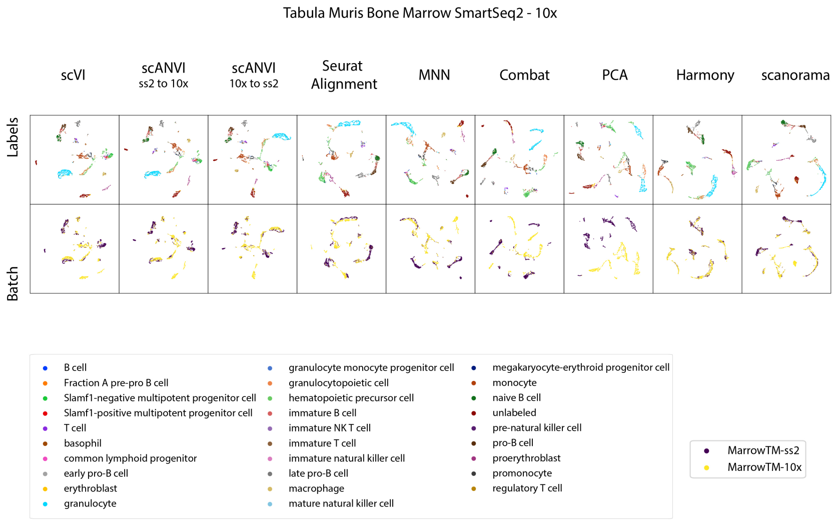

Appendix Figure 5: Visualization of the benchmark MarrowMT 10x / ss2. All positions for the scatter plots are derived using UMAP on the latent space of interest.

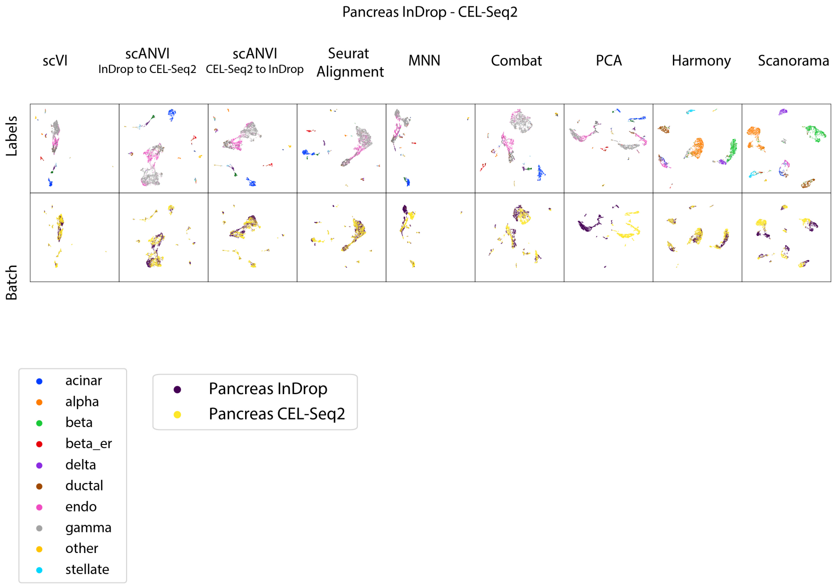

Appendix Figure 6: Visualization of the benchmark Pancreas InDrop / CEL-Seq2. All positions for the scatter plots are derived using UMAP on the latent space of interest.

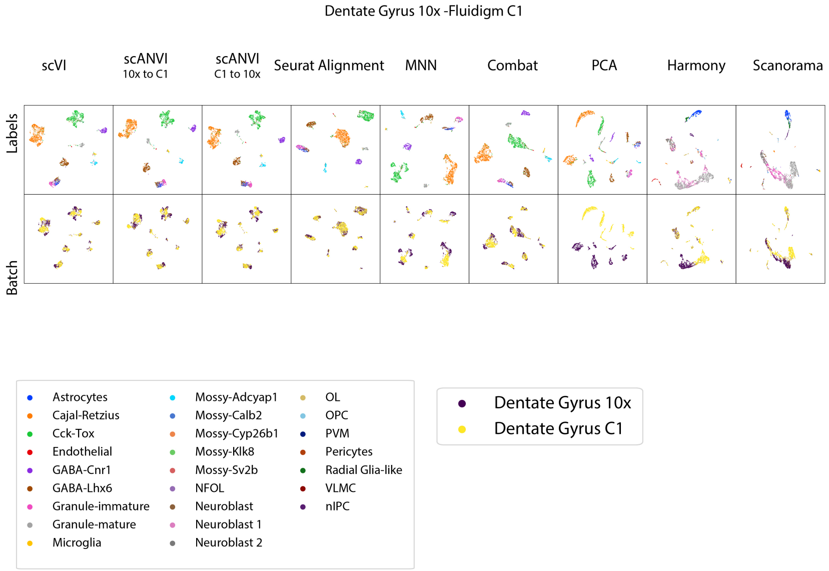

Appendix Figure 7: Visualization of the benchmark DentateGyrus10X - Fluidigm C1. All positions for the scatter plots are derived using UMAP on the latent space of interest.

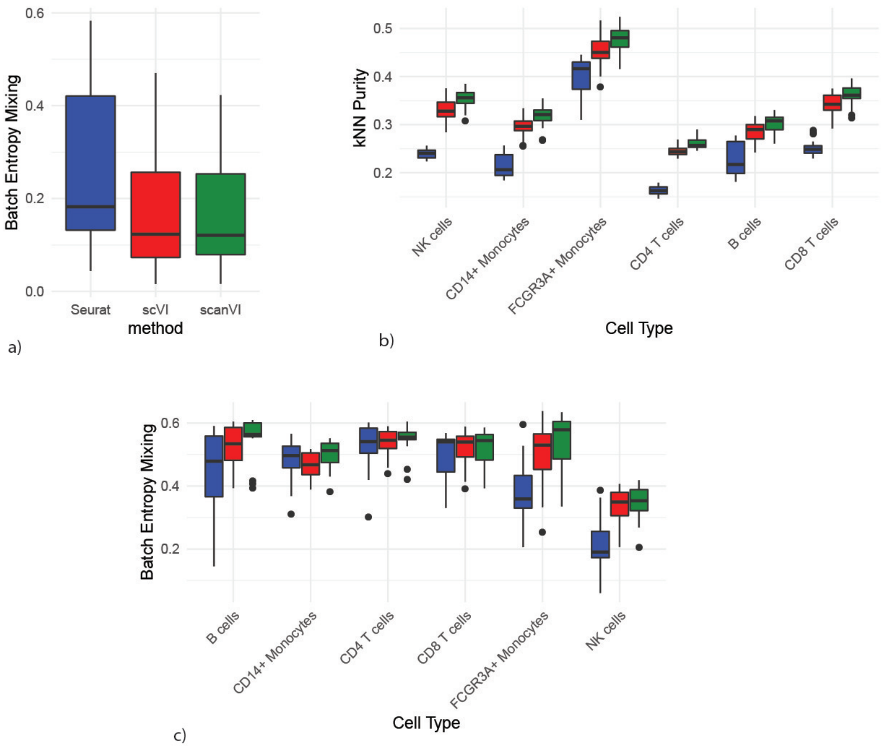

Appendix Figure 8: Supplementary study of harmonizing datasets with different cellular composition. We show here the case where each of the two datasets has a unique cell types and share all the others. For each box plot, we report over all the possible combinations of left-out cell types. (a) Entropy of batch mixing for the unique population (lower is better). (b) k-nearest neighbor purity (unique and non-unique; higher is better). (c) Entropy of batch mixing for the non-unique populations (higher is better). The boxplots are standard Tukey boxplots where the box is delineated by the first and third quartile and the whisker lines are the first and third quartile plus minus 1.5 times the box height. The dots are outliers that fall above or below the whisker lines.

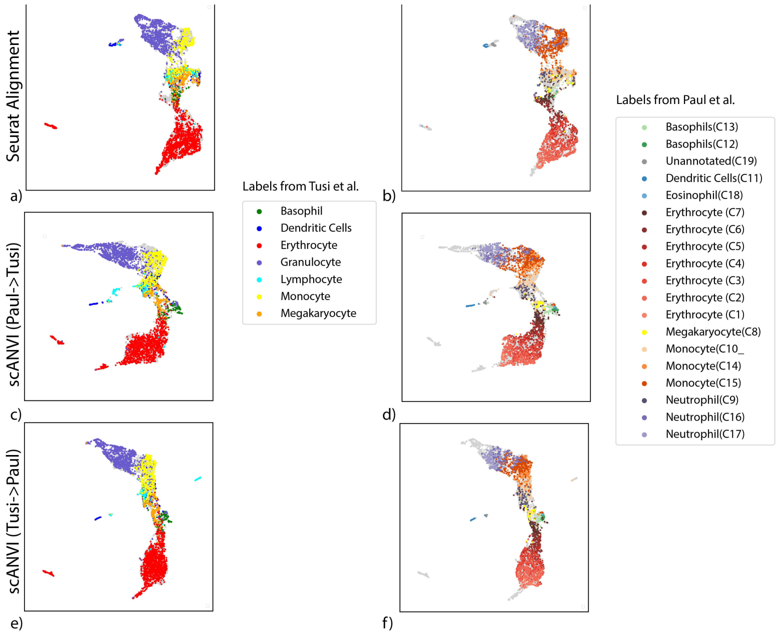

Appendix Figure 9: Follow-up analysis on continuous trajectory harmonization with scANVI. $(a-b)$ Continuous trajectory obtained by the Seurat Alignment procedure for the HEMATO-Tusi and the HEMATO-Paul datasets. $(c-d)$ Continuous trajectory obtained by the scANVI using the Tusi cell type labels for semi-supervision. $(e-f)$ Continuous trajectory obtained by the scANVI using the Paul cluster labels for semi-supervision. All locations for scatter plots are computed via UMAP in their respective latent space.

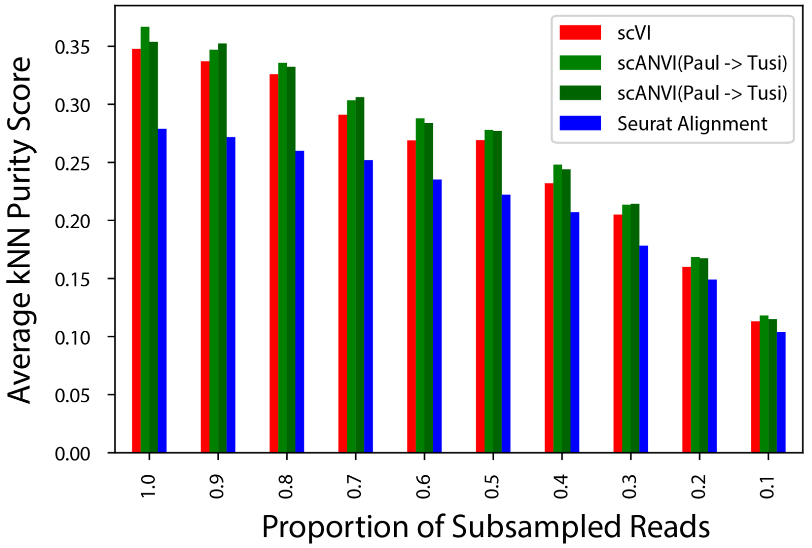

Appendix Figure 10: Average kNN purity by scVI, scANVI and Seurat Alignment when lower quality data is simulated by downsampling to 10-90% of the original transcript counts. 10% of the reads are removed from the dataset at each step, and the change in average kNN Purity score is shown on the y-axis.

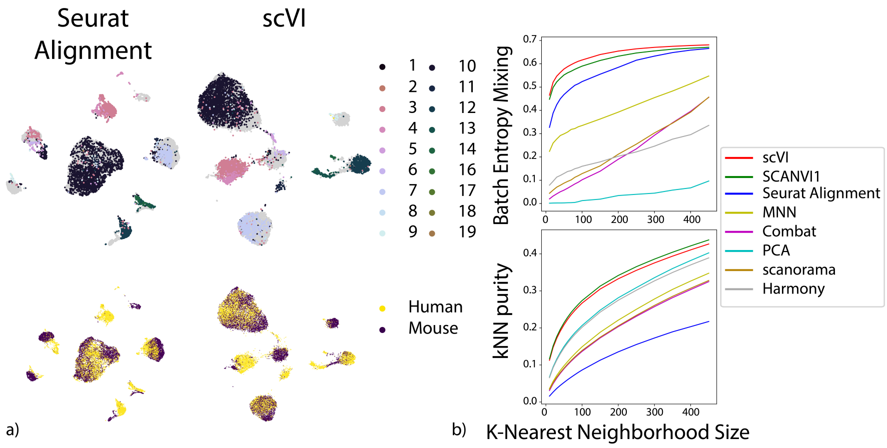

Appendix Figure 11: The harmonization performance of scVI and scANVI on datasets from human and mouse Substantia Nigra. (a) shows the distribution of mouse cell clusters and species origin on scVI and Seurat alignment latent space visualized by UMAP. The mouse cell cluster ids are provided by the original publication. (b) shows kNN purity and Batch Entropy Mixing of different methods on the cross species comparison as a function of the K-Nearest Neighborhood size.

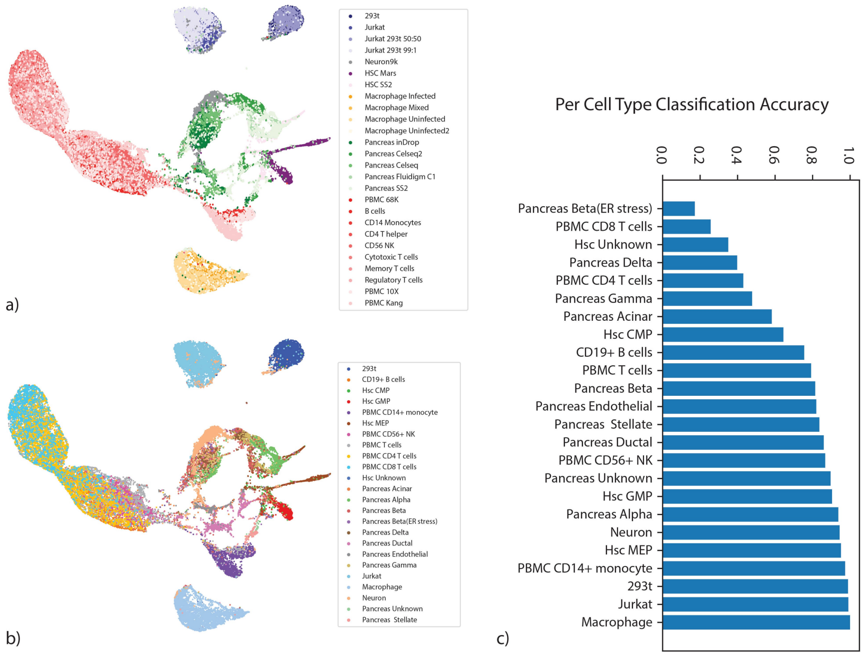

Appendix Figure 12: Large-scale data integration with scVI. $(a-b)$ UMAP visualization of the scVI latent space colored by datasets (a) and by cell types (b). (c) accuracy of a nearest neighbor classifier based on scVI latent space

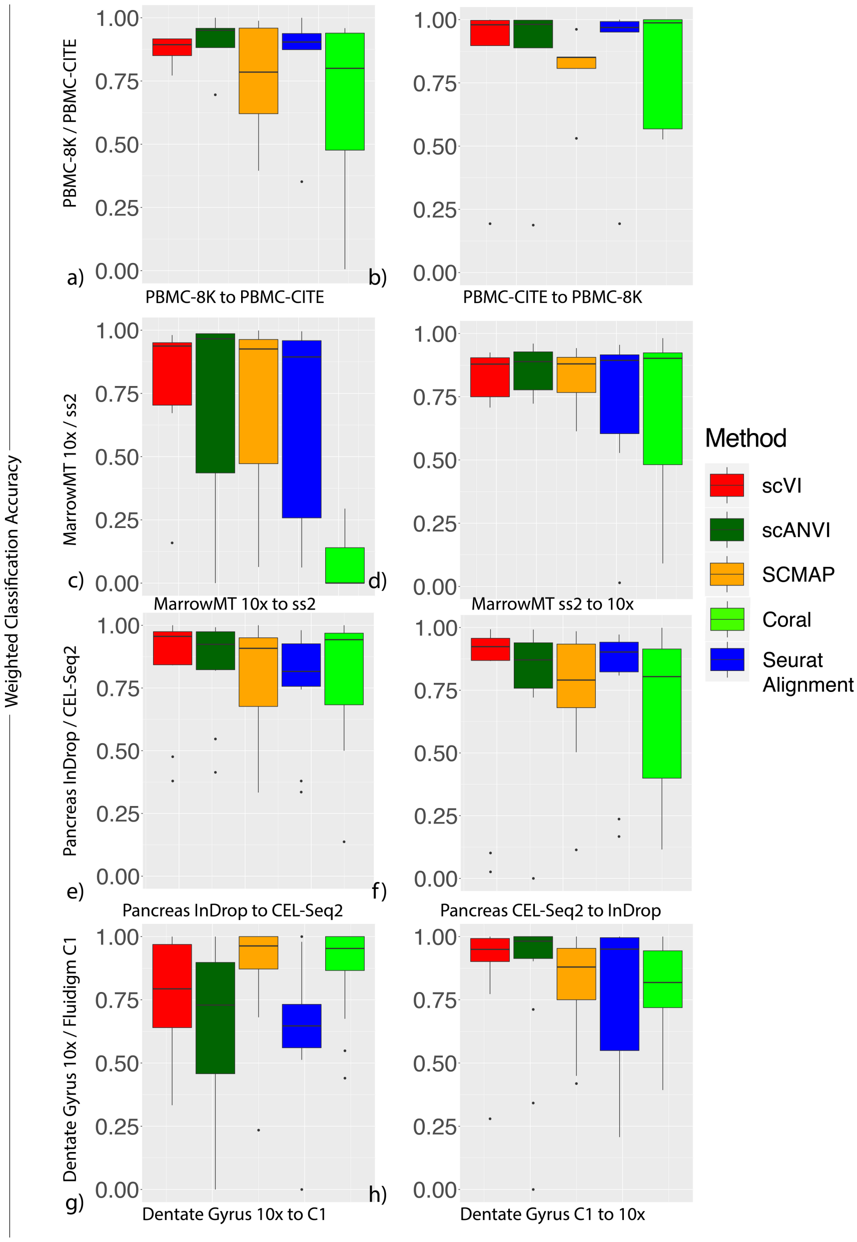

Appendix Figure 13: Annotation results for all four dataset pairs. PBMC-8K / PBMC-cite $(a-b)$, MarrowMT-10X / MarrowMT-SS2 $(c-d)$, Pancreas InDrop-CELSeq2 $(e-f)$ and Dentate Gyrus 10X / Fluidigm C1 $(g-h)$. Accuracies for transferring annotations from one dataset to another from a k-nearest neighbors classifier on Seurat Alignment, and scVI latent space, scANVI, SCMAP and CORAL classifier are shown. The aggregated results across for cell types that are shared between the two datasets is shown in box plots.

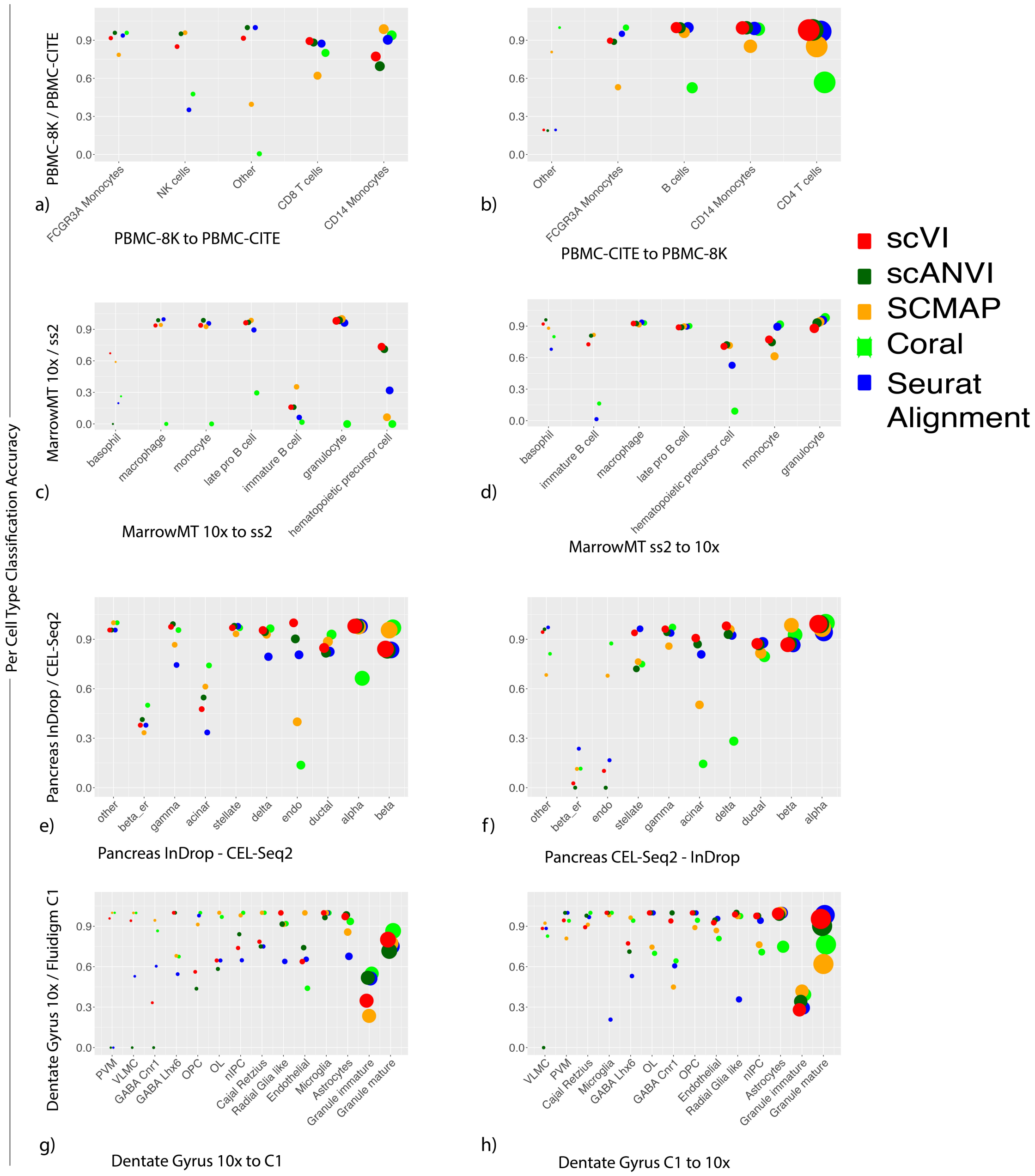

Appendix Figure 14: Annotation results for all four dataset pairs. PBMC-8K / PBMC-cite $(a - b)$, MarrowMT-10X / MarrowMT-SS2 $(c - d)$, Pancreas InDrop-CELSeq2 $(e - f)$ and Dentate Gyrus 10X / Fluidigm C1 $(g - h)$. Accuracies for transferring annotations from one dataset to another from a k-nearest neighbors classifier on Seurat Alignment, and scVI latent space, scANVI, SCMAP and CORAL classifier are shown. The prediction accuracy for each cell type that is shared between the two datasets is shown on the y-axis and the size of the dots are proportional to the proportion of a cell type in the total population.

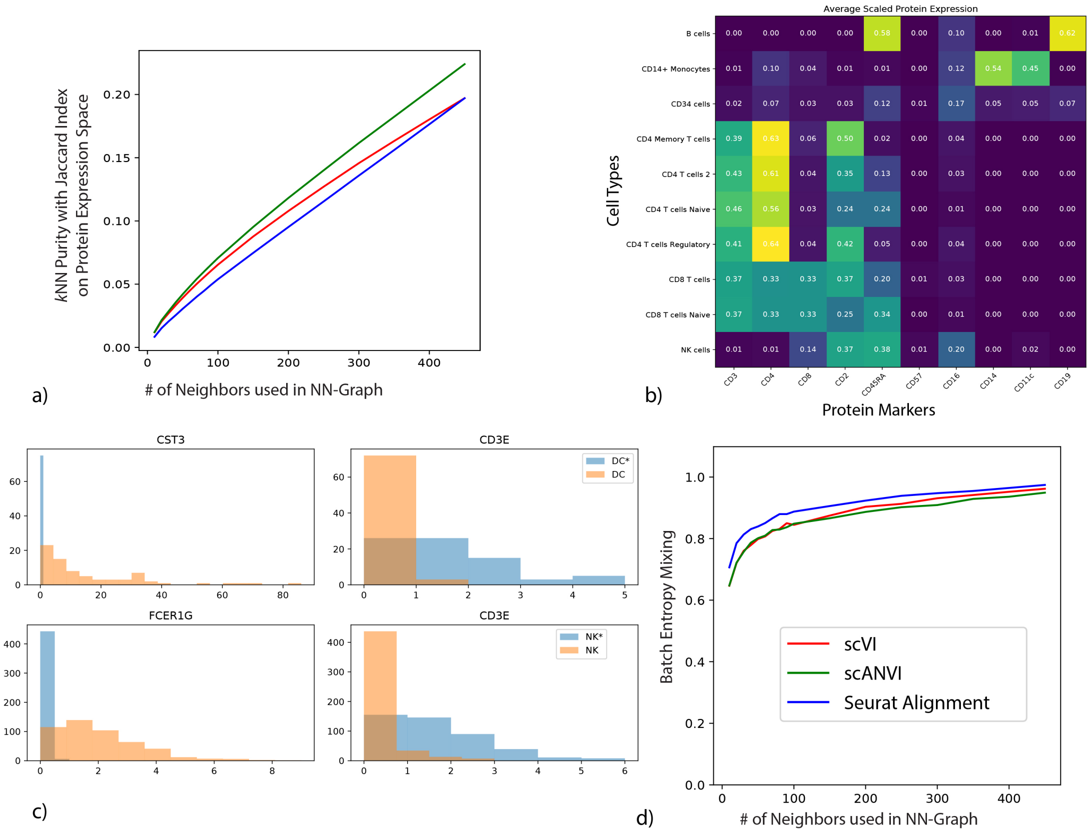

Appendix Figure 15: Supplementary study of labels concordance. (*a*) k-nearest neighbors purity of the merged latent space on the protein expression space as a function of the size of the neighborhood. (*b*) Protein expression heatmap showing consistency of PBMC-Sorted labels and protein expression in PBMC-CITE. The protein expression per cell type is based on k-nearest neighbors imputation from the harmonized latent space obtained from scANVI trained with pure population labels. (*c*) We select individual cells that were labeled as dendritic cells or Natural Killer cells in the original publication of the respective datasets, and compare the raw transcript count from cells inside the scANVI T cells cluster (DC*, NK*) against cells outside the T cells cluster (DC, NK). The expression of marker genes suggest that DC* and NK* is more likely to be T cells and thus the scANVI latent space is more accurate. (*d*) The batch entropy mixing of the three datasets in scVI, scANVI and Seurat Alignment merged space.

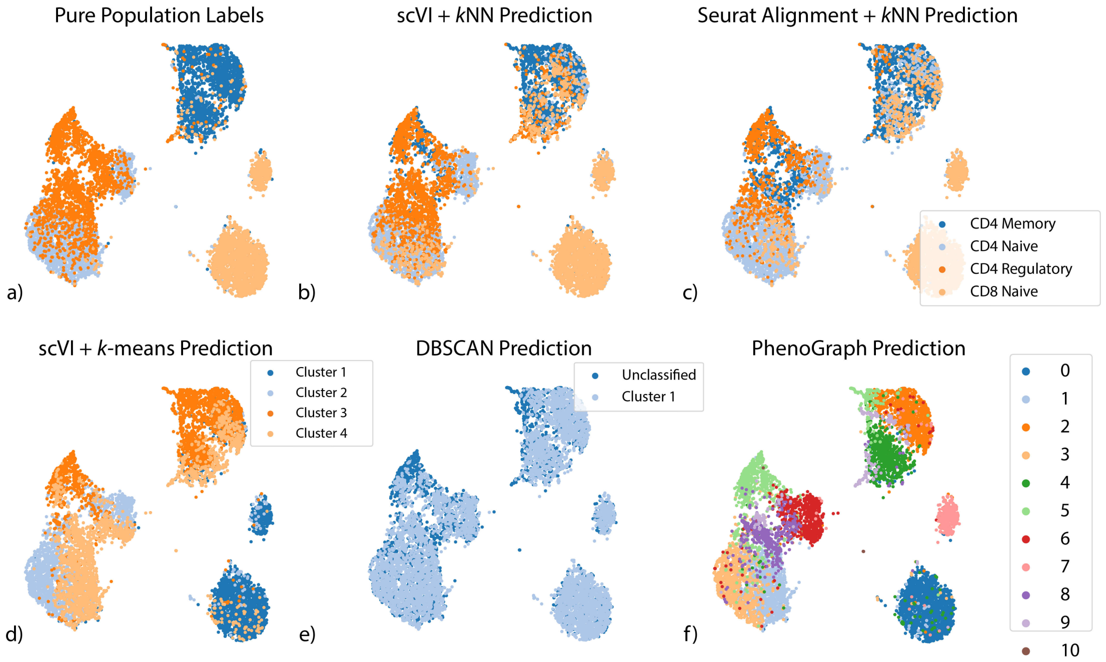

Appendix Figure 16: Other methods of classifying T-cell subsets of the PBMC-Pure dataset. Coordinates for the scatter plots are derived from UMAP embedding based on the latent space of scANVI. (a) Ground truth labels from the purified PBMC populations (b) k-nearest neighbors classification labels when applied on scVI latent space from the seed set of cells (c) k-nearest neighbors classification labels when applied on Seurat Alignment latent space (d) k-means clustering based labels when applied to scVI latent space (e) DBSCAN clustering based labels when applied to scVI latent space. DBSCAN returns only one cluster but return some cells as unclassified. (f) PhenoGraph clusters on scVI latent space

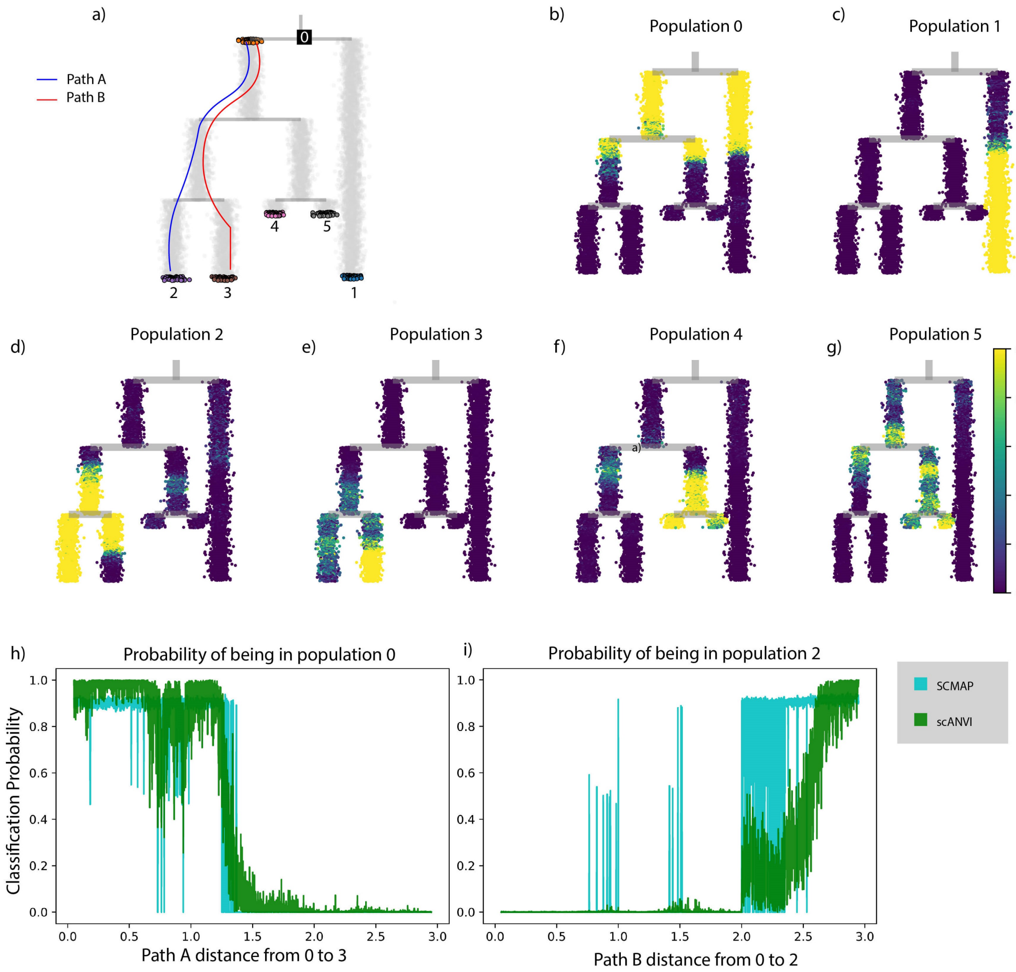

Appendix Figure 17: Continuous trajectory simulated using SymSim. (a) Tree structure from which the cells are sampled. Each grey dot represent a cell sampled along the trajectory. Colored dots with a black edge are treated as labeled, while the others are treated as unlabeled. Each path simulates a continuous phenotypical variation. $(b-g)$ The same tree with each cell colored by the posterior probability of being assigned to a specific label. $(h-i)$ Another visualization of the gradual change of posterior probability by plotting the posterior probability of root (h) and population 3 (i). The x-axis represents the pathwise distance (paths are defined in (a)), and the y-axis represents the probability, or confidence of the assignment.

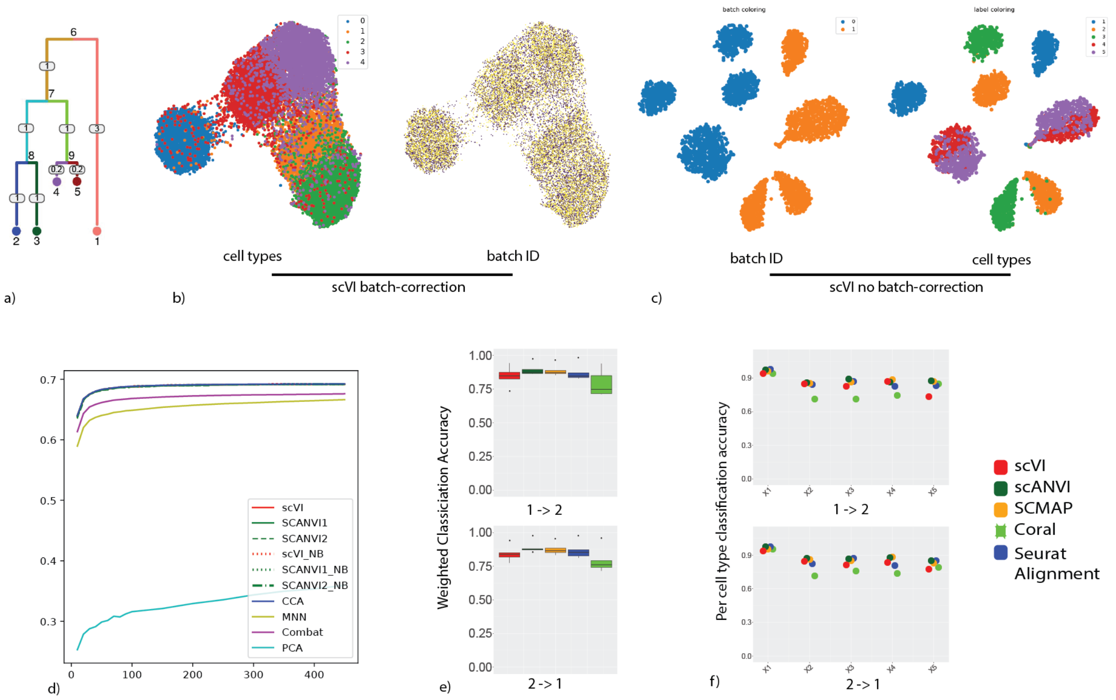

Appendix Figure 18: Presentation of the simulated dataset used for differential expression benchmarking. (a) The tree used to sample the cells in SymSim. We sample cells from the five leaves nodes representing five different cell types derived from the same root node. (b) UMAP of scVI latent space colored by cell types and batch identifier (c) UMAP of scVI latent space without batch correction, proving that the data is indeed subject to batch effects. (d) Entropy of batch mixing for all the algorithms (e) Weighted accuracy using a k-nearest neighbors classifier on the latent space (f) Per cell type accuracy for the label transfer.

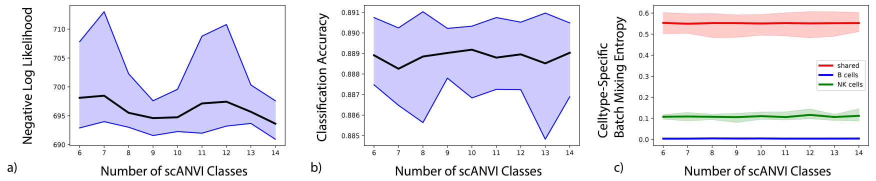

Appendix Figure 19: The effect of the choice of number of classes on the scANVI model likelihood (a), classification accuracy (b) and entropy of batch mixing (c). We trained scANVI using PBMC8K as the labelled dataset, and varied the number of classes in scANVI from 6(true number of labelled cell types) to 14. The thicker line show the mean of 9 replicates, while the colored shading show the 95% confidence interval. We used a subsampled PBMC8K-CITE dataset, where NK cells are removed from the PBMC8K dataset and B cells are removed from the PBMC-CITE dataset. As we expect, the two unique dataset have low mixing in (c) while the other cell types have high mixing. Although there is no labelled B cells, scANVI does not cluster B cells from the PBMC8K dataset with other cell types in PBMC-CITE. The three metrics we use to evaluate scANVI performance are minimally affected by the increase in the number of classes.

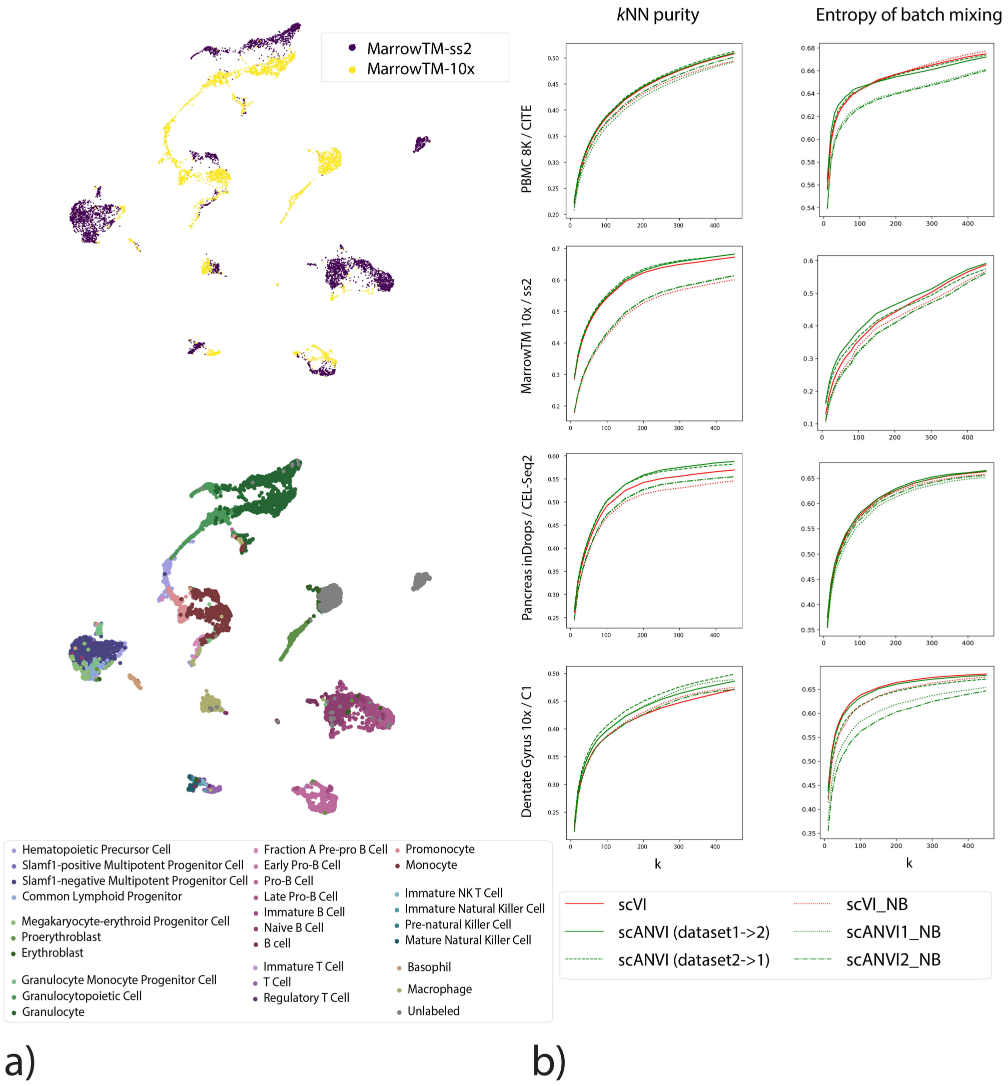

Appendix Figure 20: Performance of scVI and scANVI with a negative binomial (NB) distribution. (*a*) UMAP plot of the MarrowTM pair using a NB distribution for scVI. (*b*) Harmonization statistics and differences between regular scVI and NB version (scVI-NB). Dotted lines represent results using scVI-NB.

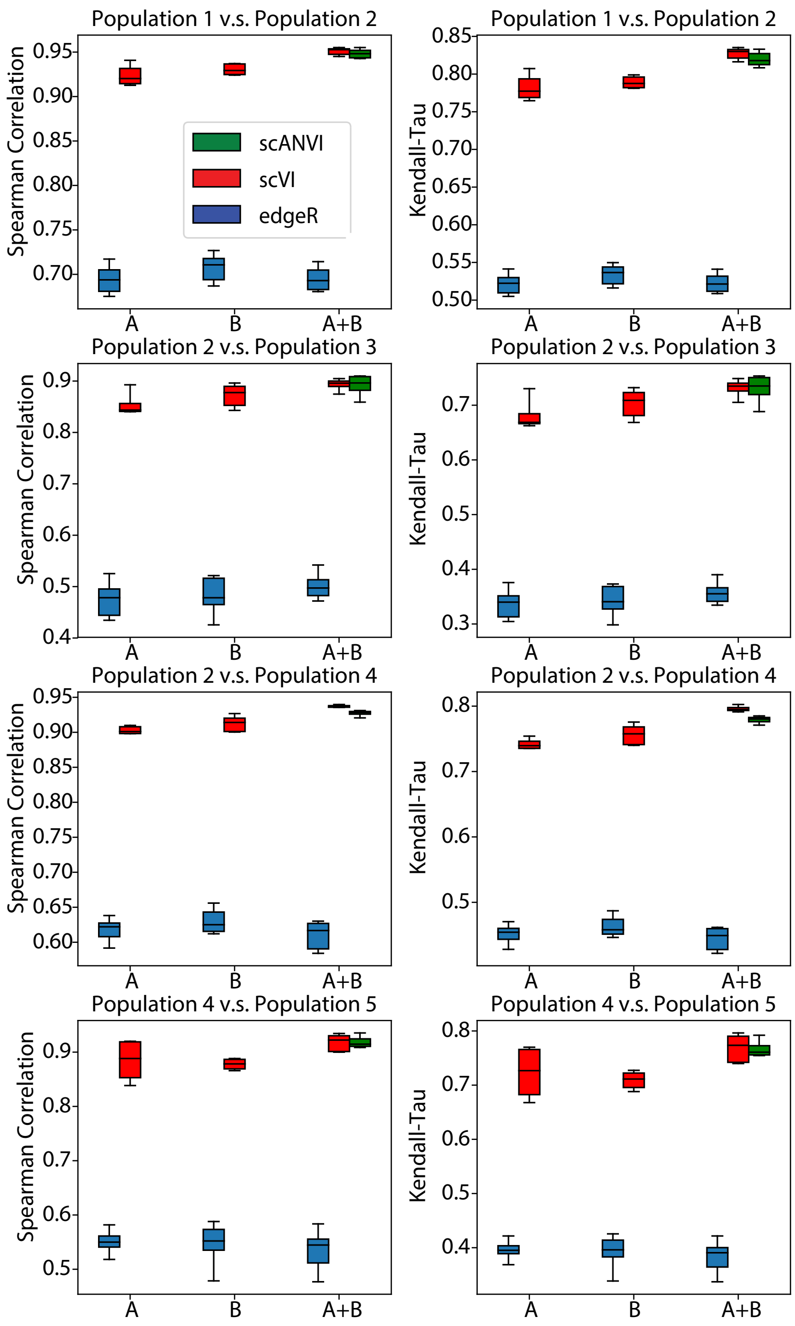

Appendix Figure 21: Differential Expression with a negative binomial version of scVI. We report all metrics on all pairs of cell types using the simulated dataset previously analyzed as in **Appendix Figure Appendix Figure 18a**.

Appendix Tables

Dataset Name	Tech.	n cells	Description	Ref.
**PBMC-8K**	10x	8,381	peripheral blood mononuclear cells (PBMCs) from a healthy donors; labels extracted from [Cole et al., 2019]	[10x, 2017]
**PBMC-CITE**	10x	7,667	PBMCs obtained from CITE-seq; labels generated manually by inspection of protein marker level on Seurat clusters	[Stoeckius et al., 2017]
**PBMC-68K**	10x	68,579	fresh PBMCs collected from healthy donor	[Zheng et al., 2017]
**PBMC-Sorted**	10x	94,655	Bead-purified PBMCs collected from the same donor as PBMC68K	[Zheng et al., 2017]
**MarrowTM-10x**	10x	4,112	Mouse bone marrow cells collected from two female mice	[Schaum et al., 2018]
**MarrowTM-ss2**	Sma.-Seq2	5,351	FACS sorted cells (B cells, T cells, granulocytes and Kit (+), Sca-1 (+) and Lin (-) hematopoietic stem cells) from 3 male and 2 female mice,	[Schaum et al., 2018]
**Pancreas-InDrop**	inDrop	8,569	Human Pancreas	[Baron et al., 2016]
**Pancreas-CELSeq2**	CEL-Seq2	2,449	Human Pancreas	[Muraro et al., 2016]
**DentateGyrus-10x**	10x	5,454	Mouse Dentate Gyrus	[Hochgerner et al., 2018]
**DentateGyrus-C1**	Fluid. C1	2,303	Mouse Dentate Gyrus	[Hochgerner et al., 2018]
**CORTEX**	10x	160,796	Mouse Nervous System	[Zeisel et al., 2018]
**HEMATO-Tusi**	inDrop	4,016	Hematopoeitic Progenitor Mouse Cells	[Tusi et al., 2018]
**HEMATO-Paul**	MARS-seq	2,730	Hematopoeitic Progenitor Mouse Cells	[Paul et al., 2015]
**SCANORAMA**	Mixture	105,476	human cells from 26 diverse scRNA-seq experiments across 9 different technologies	[Hie et al., 2019]
**SN-human**	10x	10,000 (sub-sampled)	Brain cells from human Substantia Nigra	[Welch et al., 2019]
**SN-mouse**	Drop-seq	10,000 (sub-sampled)	Brain cells from mouse Substantia Nigra [Saunders et al., 2018]	

Appendix Table 1: List of dataset used in this paper. Note that for the PBMC-Sorted 11 cell types were collected according to the paper but only 10 are available from the 10x website [10x, 2017].

Method	PBMC8KCITE	MarrowTM	Pancreas	DentateGyrus
**scVI**	0.73395	0.74325	0.81425	0.5418
**scANVI1**	0.74465	0.7418	0.76625	0.55875
**scANVI2**	**0.8184**	**0.77825**	0.78815	0.54135
**Seurat**	0.7351	0.72095	0.6174	0.4149
**MNN**	0.7364	0.6783	0.76205	0.4296
**PCA**	0.59895	0.6089	0.5474	0.4179

Appendix Table 2: Additional metric for retainment of structure via k-means clusters preservation. For scANVI we perform semi-supervision using the cell type label (not k-means cluster labels) from only one of the two datasets. Thus we train two separate models SCANVI1 and SCANVI2. To obtain a measure of clustering conservation, we first run k-means clustering in the latent space of dataset 1, then in the harmonized latent space using only cells from dataset 1. We compute the adjusted Rand Index of the two clustering results. We then do the same for dataset 2 and the final score is the average for both datasets.

cell-type	PBMC-8K proportion	PBMC-CITE proportion
**NK cells**	0.036	0.178
**CD8 T cells**	0.119	0.091
**B cells**	0.133	0.104
**FCGR3A+ Monocytes**	0.028	0.029
**CD14+ Monocytes**	0.186	0.159
**CD4 T**	0.421	0.436
**Dendritic Cells**	0.026	0
**Megakaryocytes**	0.008	0
**Other**	0.043	0.004

Appendix Table 3: Composition of cell-types in the PBMC-8K and the PBMC-CITE dataset

	Dentate Gyrus				
**ngenes**	**733**	**1,527**	**3,146**	**6,100**	**10,665**
**Seurat**	13.7	20.7	26.5	46.0	78.7
**PCA**	1.6	1.6	1.7	1.8	1.7
**scVI**	223.8	241.9	250.4	268.4	281.2
**scANVI1**	126.9	137.4	158.9	169.1	275.4
**scANVI2**	59.6	66.2	76.2	81.3	130.6
**SCMAP**	52.1	51.6	53.1	66.7	69.6
**MNN**	154.7	273.9	591.4	1141.6	2060.3
**Combat**	11.4	7.8	26.2	33.2	52.7
**scanorama**	50.3	29.1	37.5	61.7	37.8
**Harmony**	13.0	6.2	11.1	10.5	6.5

	MarrowTM				
**ngenes**	**876**	**1,731**	**3,407**	**6,546**	**11,224**
**Seurat**	21.9	34.5	42.4	71.6	123.4
**PCA**	2.0	2.0	2.0	2.0	2.1
**scVI**	289.4	290.5	298.0	305.1	419.6
**scANVI1**	159.5	151.2	162.2	178.5	236.7
**scANVI2**	115.3	129.5	127.8	143.0	187.5
**SCMAP**	74.8	78.5	82.5	84.9	134.1
**MNN**	410.6	769.1	1424.9	2471.2	4082.2
**Combat**	6.3	11.0	21.4	44.9	100.6
**scanorama**	48.0	50.9	78.9	66.2	105.5
**Harmony**	20.6	20.3	21.0	20.9	27.8

	PBMC8KCITE				
**ngenes**	**664**	**1309**	**2699**	**5623**	**10352**
**Seurat**	36.8	63.0	67.2	111.6	186.0
**PCA**	3.3	3.1	3.0	3.0	3.2
**scVI**	409.0	441.0	421.3	458.6	498.7
**scANVI1**	208.6	298.5	230.2	254.9	293.5
**scANVI2**	182.0	172.8	245.0	214.5	235.3
**SCMAP**	48.8	50.9	49.1	56.3	64.5
**MNN**	866.4	1412.1	1547.8	4915.6	8644.6
**Combat**	12.6	11.9	17.1	36.9	61.4
**scanorama**	39.5	49.9	48.1	51.8	58.6
**Harmony**	11.4	11.5	10.6	12.9	13.3

	Pancreas				
**ngenes**	**688**	**1,346**	**2,674**	**5,326**	**10,481**
**Seurat**	23.3	33.5	38.8	61.9	109.1
**PCA**	2.2	2.2	2.3	2.3	2.2
**scVI**	324.6	332.7	340.6	350.4	346.9
**scANVI1**	211.4	210.0	243.9	239.1	260.6
**scANVI2**	182.0	172.8	245.0	214.5	235.5
**SCMAP**	75.9	73.0	75.5	78.3	87.0
**MNN**	211.2	436.5	721.2	1335.2	2790.0
**Combat**	14.3	15.0	27.2	37.0	66.8
**scanorama**	52.7	54.2	55.1	58.6	60.0
**Harmony**	24.9	25.4	25.3	25.7	25.7

Appendix Table 4: Runtime in seconds for all the algorithms considered in this study.

Cell Types	# cells
**B cells**	10,085
**CD14+ Monocytes**	2,612
**CD34+ cells**	9,232
**CD4 T cells**	11,213
**CD56 NK cells**	8,385
**CD8 T cells**	10,209
**Memory T cells**	10,224
**Naive CD8 T cells**	11,953
**Naive T cells**	10,479
**Regulatory T cells**	10,263

Appendix Table 5: Cell types present in the PBMC-sorted dataset.

A Related work

A.1 Related machine learning litterature

Our approach relates to the machine learning literature in two major aspects. First, we will relate the problems of harmonization and annotation to the litterature of domain adaptation or covariate shift. Second, we will present all the different options for performing semi-supervised learning with variational autoencoders (VAEs) and further explain how they relate to scANVI.

A.1.1 Domain adaptation

In the supervised learning framework, data is drawn from a distribution $\mathbb{P}_X$ and one posits a conditional distribution $\mathbb{P}_{Y|X}$ from which to draw the labels. There are multiple flavors of domain adaptations [Kouw and Loog, 2019]. We focus here in the setting where one observes paired data on a certain source domain $\mathbb{P}_X^S\mathbb{P}_{Y|X}$ but no labels on a target domain $\mathbb{P}_X^T$ with $\mathbb{P}_X^S \neq \mathbb{P}_X^T$ (commonly referred to as distribution shift, or *covariate shift* in the statistical literature). Our problem of single-cell annotation is much related to this variant of supervised domain adaptation

where one observes the cell labels for one dataset and wishes to transfer it to another (annotated) dataset. This is a well-established research area in computer vision, where algorithms such as NBNN [Tommasi and Caputo, 2013] and JDA [Long et al., 2013] are used to transfer labels over datasets of images (e.g., with different lightning conditions, collections of images). As these algorithms might not scale to millions of samples, a notable contribution is the DANN framework [Ganin et al., 2016] which learns a adversarial classifier to in order to generalize to the new (unlabelled) dataset. This approach is justified from the statistical theory perspective as the adversarial regularization is equivalent to controlling for the $\mathcal{H}$-divergence between source domain $\mathbb{P}_X^S$ and target domain $\mathbb{P}_X^T$. Another notable contribution is the variational fair autoencoder, which focuses on semi-supervised domain adaptation with VAEs [Louizos et al., 2016] by adding a maximum mean discrepency [Gretton et al., 2012] loss between source and target latent distributions.

Moving away from the supervised domain adaptation scenario, recent work based on generative adversarial networks [Goodfellow et al., 2014] focuses on unsupervised domain adaptation of *unpaired* datapoints. This problem is relevant to the scRNA-seq methodology since one never gets to observe the same cell several times (the protocol is a destructive process). CycleGAN [Zhu et al., 2017] is based on the idea of cycle consistency (a translator that would transform a french sentence into english and then back to french again should be the identity map). This idea was then improved by Cycada [Hoffman et al., 2018], which adds more consistency to the objective function. StarGAN [Choi et al., 2018] extended CycleGAN to multiple domains while reducing the overall complexity. Finally, MAGAN [Amodio and Krishnaswamy, 2018] proposed to add a correspondence loss to further orient the manifold alignment and facilitate the inference. Notably, MAGAN [Amodio and Krishnaswamy, 2018] was also applied to merging scRNA-seq and CyTOF data.

A.1.2 Semi-supervised learning with variational autoencoders

Extending scVI to semi-supervised learning took some design that we describe here. Our first attempt was based on the M2 model [Kingma et al., 2014], and is still implemented in our codebase[1]. While this algorithm performed a posteriori as good or better than the final version of scANVI (in terms of cross-validation estimate of the cell type prediction accuracy), it restrained our latent space z to be conditioned on the cell type label (as in the conditional VAE [Sohn et al., 2015]) and it was not possible anymore to visualize the latent space, which is an crucial practice in scRNA-seq data analysis [Becht et al., 2018]. We therefore turned to extending scVI based on the M1 + M2 model [Kingma et al., 2014], which enabled both a flexible modeling of cell types and visualization of a joint latent space for all cells. We also tried more complicated models based on the M1 + M2 model such as ADGM [Maaløe et al., 2016] and LVAE [Rasmus et al., 2015] but did not find them to contribute significantly enough to the accuracy, which might be either due to the labeling errors in the dataset, because dividing cells into cell types is a relatively easy problem in most regimes (e.g., not considering rare cell types) or because of the limited sample size in the datasets we consider.

A.2 Related scRNA-seq harmonization work

A.2.1 Why is harmonization a subtle problem?

Harmonization is a hard and ill-defined problem. Especially, it can be difficult to formulate exactly its objective and at which level of granularity the “harmonization” is expected. Let us take the example of two scRNA-seq datasets of peripheral blood mononuclear cells. If we assume that these datasets that are exact biological replicates and with the same experimental conditions, then the problem is well defined. We wish to identify latent variables that govern these biological processes (for example, clear demarcation of cell types). Fundamentally, this is possible because we made a non-confounder assumption, and therefore, removing in a principled way all the variation in the data that corresponds to batches is reasonable.

However, if we consider a more complex although also more common case, the biology might be slightly different from one dataset to another. For example, in the case of T cell activation (one stimulated sample and one control sample), we expect that the overall clusters should stay similar. At a broader level, CD4 T cells should cluster together, as for all the other cell types.

[1] VAEC: `https://github.com/YosefLab/scVI/blob/master/scvi/models/vaec.py`

However, a non-negligible proportion of T cells will express markers of activation. In this case, forcing the latent spaces to exactly overlay might be problematic in the sense that this subtle signal of activation will be lost. One might think about the activation signal as a confounding factor for harmonization, and this makes the overall problem much more difficult (ill-posed).

Therefore, it is extremely important to state how different models perform harmonization and what are their underlying hypotheses and modeling capabilities.

A.2.2 State-of-the-art approaches

scmap [Kiselev et al., 2018] proposes a new gene filtering method to select gene that are claimed to be invariant to batch-effects. However, it is clear that over filtering can lead to ignoring biological information. MNN [Haghverdi et al., 2018] assumes that the topology of cell types can be easily resolved by a k-nearest neighbors graph, where neighborhoods are defined with respect to the cosine distance. This allows MNN [Haghverdi et al., 2018] and all the neighbor-matching-based approaches [Stuart et al., 2019, Hie et al., 2019] to remarkably merge batches with the risk of also merging cell types in the case where they are not detectable with this normalization scheme. SAUCIE [Amodio et al., 2019] and MMD ResNet [Shaham et al., 2017] both propose to perform batch-correction by adding on their objective function a non-parametric measure of distance between distributions (maximum mean discrepancy). This approach specifically assumes that each dataset has the same cell type composition and the same biological signal. Therefore, SAUCIE and ResNet are susceptible to perform over-correction. Seurat Alignment [Butler et al., 2018] relies on a milder assumption that there is a common signal exactly reproducible between the two datasets and that CCA capture most of the biological variation. This is not obviously true considering limited suitability of linear Gaussian model for scRNA-seq. Seurat anchors [Stuart et al., 2019] relies on CCA and suffers from the same problems as MNN with its specific normalization scheme. Finally, the recent LIGER [Welch et al., 2019] method at first sight seems like a non-probabilistic version of scVI since it also learns a degenerate conditional distribution via its integrative non-negative matrix factorization [Welch et al., 2019] (NMF is a noisy-less version of a Gamma-Poisson generative model). However, it has a further quantile normalization of the latent spaces within clusters. First, the output of the clustering algorithm is not necessarily correct and could perturb downstream analyses. Second, if a cell type would be slightly different from one condition to another, this information would be lost in the final latent space. Overall, all these correction methods can therefore potentially lead to over-correction and statistical artifacts [Nygaard et al., 2016].

A.2.3 The approach taken by this manuscript

scVI and scANVI perform harmonization by learning a common generative model for a collection of gene expression probability distributions $[p(x \mid z, s)]_{s \in \{1, \cdots, K\}}$ indexed by the dataset-identifier s. The statistical richness of the collection of conditional distributions dictates the flexibility of our model towards integrating datasets.

Another notable factor that sensibly contributes to harmonization capabilities is the prior for cell-specific scaling factor l_n that is dataset-specific. This helps probabilistically removing library-size caused discrepancies in the measurements and is more principled than normalization of the raw data [Vallejos et al., 2017]. Another important parameter is the parametrization of the neural network that maps variables (z, γ) to the expected frequencies $\mathbb{E}[w \mid z, s] = f_w(z, s)$. Since our function f_w is now potentially non-linear, our model can benefit from more flexibility and fit batch-specific effects locally for each cell types or phenotypical condition. Especially, depending on how one designs the neural architecture of f_w, it is possible to more flexibly correct dataset-specific effects. More specifically, we treat f_w as a feed-forward neural network for which at each layer we concatenate the batch-identifier with the hidden activations. A consequence of this design is that with more hidden layers in f_w, less parameters are shared and the family of distributions $[p(x \mid z, s)]_{s \in \{1, \cdots, K\}}$ has more flexibility to fit batch-specific effects (**Supplementary Figure Appendix Figure 2a-c**). Conversely, when the latent dimension grows, the generative network has less incentive to use the dataset covariate and might mildly duplicate the information in its latent space (**Supplementary Figure Appendix Figure 2d-f**). Throughout the paper, we have fixed those parameters (**Materials and Methods**) and show competitive performance for all our datasets.

Another insight comes from how the variational distribution $q(z \mid x, s)$ is parametrized. Our neural networks play the role of an explicit stochastic mapping from the gene expression x_n of a single-cell n to a location in a latent space z_n (a standard theme in scRNA-seq analysis). With this map, we match an empirical distribution in gene expression space (i.e, a dataset) $p_{\text{data}}(x, s) = \sum_n \delta_{(x_n, s_n)}(x, s)$, with a certain distribution on the latent space $p_{\text{data}}(z) = \sum_n \delta_{\Phi(x_n, s_n)}(z)$. In certain cases, even though we designed this latent space to represent biological signal, the transformed dataset might still be confounded by technical effects [Lopez et al., 2018]. In particular, this effect is susceptible to be severe when the generative model is not flexible enough to fit the dataset-specific effects or has a model misfit (as in, to some extent, the case with the CCA of [Butler et al., 2018] that assumes the data is log-normal). In this case, the go-to method is to empirically constrain the mapping (i.e the variational network in our case or the latent space itself in the case of SEURAT) to match the collections of variables $p_{\text{data}}(z, s)_{s \in \{1, \cdots, K\}}$. This is what is done via all the methods presented above. Therefore, our method might be improved with respect to the entropy of batch mixing by adding some specific penalties to the loss function, but at the price of risking to over-correct. We have preferred to not add any penalties, which is enough for most applications (especially all those in this manuscript).

B Training Information

In order to train scANVI properly, several options are possible to train all the parameters (θ from the generative model, ϕ from the variational distribution except the labels' posterior and $\phi^{\mathcal{C}}$ from the labels' posterior exclusively). In all cases, parameters θ and ϕ should minimize the evidence lower bound

$$\mathcal{J} = \mathcal{L} + \mathcal{U}, \tag{1}$$

decomposed over the labeled samples $\mathcal{L}$ and unlabeled samples $\mathcal{U}$. Furthermore, [Kingma et al., 2014] suggests to jointly optimize a modified objective that penalize the ELBO by a classification loss $\mathcal{C}$ on the labeled data so that the parameters $\phi^{\mathcal{C}}$ also benefits from the learned data. They introduce the modified objective function

$$\mathcal{J}_\alpha = \mathcal{J} + \alpha \cdot \mathcal{C}, \tag{2}$$

where α is a parameter set by cross-validation. This modified lower bound can be interpreted as placing a Dirac prior on c [Kingma et al., 2014] or as a correction term for noisy labels [Langevin et al., 2018]. In their procedure, $\mathcal{J}_\alpha$ is optimized with respect to all parameters $[\theta, \phi, \phi^{\mathcal{C}}]$ and for a fixed number of epochs. This joint training procedure is appealing as it shapes the latent space directly, through the modification of the encoder's weights. However, we found this approach to have two limitations. First, this joint training breaks down the mixing in the latent space in the case of transferring labels from one dataset to another. We attribute this to the loss $\mathcal{C}$ having only contributions from a unique dataset. Second, we did not find convenient to choose the optimal value for the parameter α and concluded it may not be desirable for a practitioner either.

We use in scANVI an alternate training procedure which deletes the need for α and has better performance in the setting of transferring annotations. We aggressively train the classifier separately, updating the parameters $\phi^{\mathcal{C}}$ for $c > 1$ epochs for every single epoch of updating $[\theta, \phi]$. The total number of epochs is fixed and chosen high enough to guarantee convergence. In the case of a single dataset (resp. transfer of labels), we use $c = 1$ (resp. $c = 100$) epochs of classifier training in between each variational update. This helps the classifier correctly identify cell types at the end of each epoch of updating $\phi^{\mathcal{C}}$. This is a clearly advantageous procedure, because it then improve indirectly the latent space quality, through the next steps of optimization.

C Alternative model choices

C.1 Zero-inflation in the context of harmonization

In this manuscript, we mainly rely on a zero-inflated negative binomial (ZINB) distribution for the counts — which is a widely used model for single cell transcriptomics data. Recent research however suggests that for some technologies such as droplet-based UMI single-cell protocols,

the abundance of zero mainly may be explained only by limited sensitivity and subsampling effect [Svensson, 2019]. On the other hand, zero-inflated might be a realistic addition to count distributions for describing full-length plate-based technologies data [Vieth et al., 2017]. Indeed, it has been hypothesized that PCR duplication or uneven fragment sampling may be responsible for zero-inflation in plate-based technologies [Svensson, 2019]. Still, no definite conclusion can be drawn about which distribution better fits UMI technologies or full-length method. Although full-length methods seem more affected by technical noise, they also provide extra information at the transcript resolution that is lost in droplet-based UMI methods. Consequenly, many consortia have chosen to obtain data by both full-length and UMI methods [Schaum et al., 2018, Ecker et al., 2017]. A method that can flexibly use different distribution and appropriately these two data types is therefore extremely valuable for analyzing collections of single cell transcriptomics data.

scVI and scANVI are both flexible to use both ZINB and NB methods and we show in **Supplementary Figure Appendix Figure 20** that the benchmarking results are similar using both methods in all four datasets used in our benchmarking procedure. However, we observed that for MarrowTM dataset, using the ZINB version of scVI does perform better in terms of harmonization. Since in this case, we are merging a 10x dataset with a Smart-Seq2 dataset, we compared the average zero-inflation parameter for each gene in each dataset and found that the differences between Smart-Seq2 and 10x are significantly skewed to the right (more zero-inflation in Smart-Seq2, p=6.3e-31, using a D'Agostino-Pearson K^2 test). Finally, we also performed differential expression with negative binomial versions of scVI and scANVI and show that results are similar (**Supplementary Figure Appendix Figure 21**). These results show that both NB and ZINB model can be used in scVI and scANVI and in most instances, downstream analysis might not be impacted by this modeling choice. Interestingly, our model successfully learns the difference in the degree of zero-inflation in different datasets while merging them and exploits this information when necessary.

C.2 Library-size prior for scVI and scANVI

Besides zero-inflation, another major difference between sequencing technologies is the sequencing depth. scVI and scANVI both make use of technical scalar factors that have a batch-specific prior, and are therefore extremely suited for this setting. To evaluate the effectiveness of both the model and the prior choice, we compute the negative log likelihood of the scVI model using ZINB with batch-specific library size prior, ZINB with shared library size prior, NB with a batch-specific library size prior, and NB with shared library size prior. All four models are fit on two pairs of datasets, DentateGyrus (Fluidigm C1 and 10x) and MarrowTM (10x and SmartSeq2). Since only the second pair is a comparison between UMI and full-length datasets, we expect the differences between the four model choices to be larger in the MarrowTM comparison, and that ZINB with batch-specific library size prior to have the highest likelihood (reported in the table below).

**Negative log-likelihood**	**DentateGyrus**	**MarrowTM**
**ZINB model / batch-specific prior**	514.5	2339.8
**ZINB model / shared prior**	513.8	2349.2
**NB model / batch-specific prior**	523.4	2531.3
**NB model / shared prior**	524.0	2544.1

Table: Assessing the model's fit with different configuration on two pairs of dataset

D Hierarchical classification

In this note, we explain how we extended scANVI to handle a two-level hierarchical structure for the cell types annotation. This can in principle be adapted to any arbitrary tree representation of cell types taxonomy, but is left for future work. In our setting, the taxonomy needs to be hard-coded and known *a priori*.

We do not modify the generative model but only the structure of the variable c_n in the variational distribution. Notably, we formally pose:

$$c_n = (y_n, y_n^g) \in \{0, \cdots, C\} \times \{0, \cdots, C^g\}, \tag{3}$$

where C denotes the number of cell types and C^g the number of cell type groups. The parametrization of the full variational distribution $q(c \mid z) = q(y, y^g \mid z)$ must be further defined. For this, we

notice that the prior taxonomy knowledge encapsulates whether the assignment $(y^g, y) = (i, j)$ is biologically possible (i.e cell type i is a sub-population of group cell type j). We encode this biological compatibility into a parent function $\pi : \{0, \cdots, K\} \rightarrow \{0, \cdots, K^g\}$ that maps a cell type to its parent in the hierarchy. We note for simplicity:

$$q(y_i, y_j^g \mid z) = q(y = i, y^g = j \mid z). \tag{4}$$

We then use two neural networks f and f_g (with softmax non-linearities) to map the latent space to the joint approximate posterior $q(y, y^g \mid z)$ with the following rules:

$$\begin{aligned} q(y_i, y_j^g \mid z) &= \begin{cases} f_i(z) & \text{if } \pi(i) = j, \\ 0 & \text{otherwise.} \end{cases} \\ q(y_j^g \mid z) &= f_j^g(z). \end{aligned} \tag{5}$$

Then, we can derive the marginal probability over finer cell types classes using the chain rule and Bayes rule:

$$\begin{aligned} q(y_i \mid z) &= q(y_i \mid y_{\pi_i}, z) q(y_{\pi_i} \mid z) && (6) \\ &= \frac{q(y_i, y_{\pi_i} \mid x)}{q(y_{\pi_j} \mid x)} q(y_{\pi_i} \mid z) && (7) \\ &= \frac{q(y_i, y_{\pi_i} \mid x)}{\sum\limits_{j \in c(\pi_i)} q(y_j, y_{\pi_j} \mid x)} q(y_{\pi_i} \mid z) && (8) \\ &= \frac{f_i(z)}{\sum\limits_{j \in c(\pi_i)} f_j(z)} f_{\pi_i}^g(z), && (9) \end{aligned}$$

where $c(\pi_i)$ denotes the set of children of node i children.

E Evidence Lower Bound decomposition

We drop the parameters Θ (resp. Φ) of the generative model (resp. the variational distribution) for notational convenience, as well as the conditioning on the batch identifier s. We report the evidence lower bound (ELBO) only for one sample (i.e one cell) and drop the index notations by substituting $\{x_n, z_n, u_n, c_n, l_n\}$ by $\{x, z, u, c, l\}$. This is without loss of generality since all the cells are independent and identically distributed under our model.

We derive the ELBO in the case where c is not observed (almost same calculations resolve the case where c is observed). Similar derivations can be found in the variational autoencoder literature (e.g, [Kingma et al., 2014]). We assume our variational distribution factorizes as:

$$q(c, z, u, l \mid x) = q(z \mid x) q(c \mid z) q(u \mid z, c) q(l \mid x). \tag{10}$$

In this case, we may simply apply Jensen's inequality weighted by the variational distribution $q(z, u, l, c \mid x)$:

$$\begin{aligned} \log p(x) \geq & \mathbb{E}_{q(z,u,c,l \mid x)} \left[\log \frac{p(x, z, u, c, l)}{q(z, u, c, l \mid x)} \right] = \mathbb{E}_{q(z,u,c,l \mid x)} \left[\log \frac{p(x \mid z, l) p(z \mid u, c) p(c) p(u) p(l)}{q(z, u, c, l \mid x)} \right] \\ \geq & \underbrace{\mathbb{E}_{q(z,u,c,l \mid x)} \left[\log p(x \mid z, l) \right]}_{(i)} + \underbrace{\mathbb{E}_{q(z,u,c,l \mid x)} \left[\log \frac{p(z \mid u, c)}{q(z \mid x)} \right]}_{(ii)} \\ & + \underbrace{\mathbb{E}_{q(z,u,c,l \mid x)} \left[\log \frac{p(c)}{q(c \mid z)} \right]}_{(iii)} + \underbrace{\mathbb{E}_{q(z,u,c,l \mid x)} \left[\log \frac{p(u)}{q(u \mid z, c)} \right]}_{(iv)} \\ & + \underbrace{\mathbb{E}_{q(z,u,c,l \mid x)} \left[\log \frac{p(l)}{q(l \mid x)} \right]}_{(v)} \end{aligned} \tag{11}$$

Then we simplify each individual term of the ELBO in 11 by recognizing KL divergence terms. In particular, we use subscript notation $\mathbb{KL}_{\mathrm{G}}(.||.)$, and $\mathbb{KL}_{\mathrm{M}}(.||.)$ to denote Gaussian and multinomial KL divergences.

$$\mathbb{E}_{q(z,u,c,l \mid x)}\left[\log p(x \mid z,l)\right] = \mathbb{E}_{q(z \mid x)q(l \mid x)}\left[\log p(x \mid z,l)\right] \tag{i}$$

$$\begin{aligned}\mathbb{E}_{q(z,u,c,l \mid x)}\left[\log \frac{p(z \mid u,c)}{q(z \mid x)}\right] &= \mathbb{E}_{q(z \mid x)}\left[\mathbb{E}_{q(u \mid z,c)q(c \mid z)}\left[\log \frac{p(z \mid u,c)}{q(z \mid x)}\right]\right] \\ &= \mathbb{E}_{q(z \mid x)}\left[\sum_{c=1}^{K} q(c \mid z)\mathbb{E}_{q(u \mid z,c)}\left[\log \frac{p(z \mid u,c)}{q(z \mid x)}\right]\right]\end{aligned} \tag{ii}$$

$$\mathbb{E}_{q(z,u,c,l \mid x)}\left[\log \frac{p(c)}{q(c \mid z)}\right] = \mathbb{E}_{q(z \mid x)}\left[\underbrace{\sum_{c=1}^{K} q(c \mid z)\log \frac{p(c)}{q(c \mid z)}}_{-\mathbb{KL}_{\mathrm{M}}(q(c \mid z)||p(c))}\right] \tag{iii}$$

$$\mathbb{E}_{q(z,u,c,l \mid x)}\left[\log \frac{p(u)}{q(u \mid z,c)}\right] = \mathbb{E}_{q(z \mid x)}\left[\sum_{c=1}^{K} q(c \mid z)\underbrace{\mathbb{E}_{q(u \mid z,c)}\left[\log \frac{p(u)}{q(u \mid z,c)}\right]}_{-\mathbb{KL}_{\mathrm{G}}(q(u \mid z,c)||p(u))}\right] \tag{iv}$$

$$\mathbb{E}_{q(z,u,c,l \mid x)}\left[\log \frac{p(l)}{q(l \mid x)}\right] = -\mathbb{KL}_{\mathrm{G}}\left(q(l \mid x)||p(l)\right) \tag{v}$$

References

[10x, 2017] (2017). 10x Genomics. https://support.10xgenomics.com/single-cell-gene-expression/datasets.

[Amodio and Krishnaswamy, 2018] Amodio, M. and Krishnaswamy, S. (2018). Magan: Aligning biological manifolds. In *Proc Int Conf Mach Learn*, pages 215–223.

[Amodio et al., 2019] Amodio, M., Van Dijk, D., Srinivasan, K., Chen, W. S., Mohsen, H., Moon, K. R., Campbell, A., Zhao, Y., Wang, X., Venkataswamy, M., et al. (2019). Exploring single-cell data with deep multitasking neural networks. *Nat Meth*, pages 1–7.

[Baron et al., 2016] Baron, M., Veres, A., Wolock, S. L., Faust, A. L., Gaujoux, R., Vetere, A., Ryu, J. H., Wagner, B. K., Shen-Orr, S. S., Klein, A. M., et al. (2016). A single-cell transcriptomic map of the human and mouse pancreas reveals inter-and intra-cell population structure. *Cell Syst*, 3(4):346–360.

[Becht et al., 2018] Becht, E., McInnes, L., Healy, J., Dutertre, C.-A., Kwok, I. W. H., Ng, L. G., Ginhoux, F., and Newell, E. W. (2018). Dimensionality reduction for visualizing single-cell data using umap. *Nat Biotechnol*, 37.

[Butler et al., 2018] Butler, A., Hoffman, P., Smibert, P., Papalexi, E., and Satija, R. (2018). Integrating single-cell transcriptomic data across different conditions, technologies, and species. *Nat Biotechnol*, 36:411.

[Choi et al., 2018] Choi, Y., Choi, M., Kim, M., Ha, J.-W., Kim, S., and Choo, J. (2018). Stargan: Unified generative adversarial networks for multi-domain image-to-image translation. In *IEEE Conf Comp Vision*, pages 8789–8797.

[Cole et al., 2019] Cole, M. B., Risso, D., Wagner, A., DeTomaso, D., Ngai, J., Purdom, E., Dudoit, S., and Yosef, N. (2019). Performance assessment and selection of normalization procedures for single-cell RNA-seq. *Cell Syst*, 8(4):315–328.

[Ecker et al., 2017] Ecker, J. R., Geschwind, D. H., Kriegstein, A. R., Ngai, J., Osten, P., Polioudakis, D., Regev, A., Sestan, N., Wickersham, I. R., and Zeng, H. (2017). The brain initiative cell census consortium: lessons learned toward generating a comprehensive brain cell atlas. *Neuron*, 96(3):542–557.

[Ganin et al., 2016] Ganin, Y., Ustinova, E., Ajakan, H., Germain, P., Larochelle, H., Laviolette, F., Marchand, M., and Lempitsky, V. (2016). Domain-adversarial training of neural networks. *J Mach Learn Res*, 17(1):2096–2030.

[Goodfellow et al., 2014] Goodfellow, I., Pouget-Abadie, J., Mirza, M., Xu, B., Warde-Farley, D., Ozair, S., Courville, A., and Bengio, Y. (2014). Generative adversarial nets. In *Adv Neural Inf Process Syst*, pages 2672–2680.

[Gretton et al., 2012] Gretton, A., Borgwardt, K. M., Rasch, M. J., Schölkopf, B., and Smola, A. (2012). A Kernel Two-Sample Test. *Journal of Machine Learning Research*, 13:723–773.

[Haghverdi et al., 2018] Haghverdi, L., Lun, A. T. L., Morgan, M. D., and Marioni, J. C. (2018). Batch effects in single-cell RNA-sequencing data are corrected by matching mutual nearest neighbors. *Nat Biotechnol*, 36(5):421–427.

[Hie et al., 2019] Hie, B., Bryson, B., and Berger, B. (2019). Efficient integration of heterogeneous single-cell transcriptomes using Scanorama. *Nature biotechnology*, 37(6):685–691.

[Hochgerner et al., 2018] Hochgerner, H., Zeisel, A., Lönnerberg, P., and Linnarsson, S. (2018). Conserved properties of dentate gyrus neurogenesis across postnatal development revealed by single-cell RNA sequencing. *Nat Neurosci*, 21(2):290.

[Hoffman et al., 2018] Hoffman, J., Tzeng, E., Park, T., Zhu, J.-Y., Isola, P., Saenko, K., Efros, A., and Darrell, T. (2018). CyCADA: Cycle-consistent adversarial domain adaptation. In *Proceedings of the 35th Proc Int Conf Mach Learn*, volume 80, pages 1989–1998.

[Kingma et al., 2014] Kingma, D. P., Mohamed, S., Jimenez Rezende, D., and Welling, M. (2014). Semi-supervised learning with deep generative models. In *Adv Neural Inf Process Syst*, pages 3581–3589.

[Kiselev et al., 2018] Kiselev, V. Y., Yiu, A., and Hemberg, M. (2018). scmap: projection of single-cell RNA-seq data across data sets. *Nat Methods*, 15:359.

[Langevin et al., 2018] Langevin, M., Mehlman, E., Regier, J., Lopez, R., Jordan, M. I., and Yosef, N. (2018). A deep generative model for semi-supervised classification with noisy labels. *arXiv*, abs/1809.05957.

[Long et al., 2013] Long, M., Wang, J., Ding, G., Sun, J., and Yu, P. S. (2013). Transfer feature learning with joint distribution adaptation. In *IEEE Int Conf Comp Vision*.

[Lopez et al., 2018] Lopez, R., Regier, J., Jordan, M. I., and Yosef, N. (2018). Information Constraints on Auto-Encoding Variational Bayes. *Adv Neural Inf Process Syst*.

[Louizos et al., 2016] Louizos, C., Swersky, K., Li, Y., Welling, M., and Zemel, R. (2016). The variational fair autoencoder. In *Proc Int Conf Learning Representations*.

[Maaløe et al., 2016] Maaløe, L., Sønderby, C. K., Sønderby, S. K., and Winther, O. (2016). Auxiliary deep generative models. In *Proc Int Conf Mach Learn*, pages 1445–1453.

[Muraro et al., 2016] Muraro, M. J., Dharmadhikari, G., Grün, D., Groen, N., Dielen, T., Jansen, E., van Gurp, L., Engelse, M. A., Carlotti, F., de Koning, E. J., et al. (2016). A single-cell transcriptome atlas of the human pancreas. *Cell Syst*, 3(4):385–394.

[Nygaard et al., 2016] Nygaard, V., Rødland, E. A., and Hovig, E. (2016). Methods that remove batch effects while retaining group differences may lead to exaggerated confidence in downstream analyses. *Biostatistics*, 17(1):29–39.

[Paul et al., 2015] Paul, F., Arkin, Y., Giladi, A., Jaitin, D., Kenigsberg, E., Keren-Shaul, H., Winter, D., Lara-Astiaso, D., Gury, M., Weiner, A., David, E., Cohen, N., Lauridsen, F., Haas, S., Schlitzer, A., Mildner, A., Ginhoux, F., Jung, S., Trumpp, A., Porse, B., Tanay, A., and Amit, I. (2015). Transcriptional heterogeneity and lineage commitment in myeloid progenitors. *Cell*, 163(7):1663 – 1677.

[Rasmus et al., 2015] Rasmus, A., Berglund, M., Honkala, M., Valpola, H., and Raiko, T. (2015). Semi-supervised learning with ladder networks. In *Adv Neural Inf Process Syst*, pages 3546–3554.

[Schaum et al., 2018] Schaum, N., Karkanias, J., Neff, N. F., May, A. P., Quake, S. R., Wyss-Coray, T., Darmanis, S., Batson, J., Botvinnik, O., Chen, M. B., et al. (2018). Single-cell transcriptomic characterization of 20 organs and tissues from individual mice creates a tabula muris. *Nature*, 562(7727):367.

[Shaham et al., 2017] Shaham, U., Stanton, K. P., Zhao, J., Li, H., Raddassi, K., Montgomery, R., and Kluger, Y. (2017). Removal of batch effects using distribution-matching residual networks. *Bioinformatics*, 33(16):2539–2546.

[Sohn et al., 2015] Sohn, K., Lee, H., and Yan, X. (2015). Learning Structured Output Representation using Deep Conditional Generative Models. In *Adv Neural Inf Process Syst 28*, pages 3483–3491.

[Stoeckius et al., 2017] Stoeckius, M., Hafemeister, C., Stephenson, W., Houck-Loomis, B., Chattopadhyay, P. K., Swerdlow, H., Satija, R., and Smibert, P. (2017). Simultaneous epitope and transcriptome measurement in single cells. *Nat Methods*, 14(9):865.

[Stuart et al., 2019] Stuart, T., Butler, A., Hoffman, P., Hafemeister, C., Papalexi, E., Mauck III, W. M., Hao, Y., Stoeckius, M., Smibert, P., and Satija, R. (2019). Comprehensive integration of single-cell data. *Cell*, 177(7):1888–1902.

[Svensson, 2019] Svensson, V. (2019). Droplet scrna-seq is not zero-inflated. *bioRxiv*, page 582064.

[Tommasi and Caputo, 2013] Tommasi, T. and Caputo, B. (2013). Frustratingly easy nbnn domain adaptation. In *IEEE International Conference on Computer Vision.*

[Tusi et al., 2018] Tusi, B. K., Wolock, S. L., Weinreb, C., Hwang, Y., Hidalgo, D., Zilionis, R., Waisman, A., Huh, J. R., Klein, A. M., and Socolovsky, M. (2018). Population snapshots predict early haematopoietic and erythroid hierarchies. *Nature*, 555(7694):54–60.

[Vallejos et al., 2017] Vallejos, C. A., Risso, D., Scialdone, A., Dudoit, S., and Marioni, J. C. (2017). Normalizing single-cell RNA sequencing data: challenges and opportunities. *Nat Methods*, pages 565–571.

[Vieth et al., 2017] Vieth, B., Ziegenhain, C., Parekh, S., Enard, W., and Hellmann, I. (2017). powsimr: power analysis for bulk and single cell rna-seq experiments. *Bioinformatics*, 33(21):3486–3488.

[Welch et al., 2019] Welch, J. D., Kozareva, V., Ferreira, A., Vanderburg, C., Martin, C., and Macosko, E. Z. (2019). Single-cell multi-omic integration compares and contrasts features of brain cell identity. *Cell*, 177(7):1873–1887.

[Zeisel et al., 2018] Zeisel, A., Hochgerner, H., Lönnerberg, P., Johnsson, A., Memic, F., van der Zwan, J., Häring, M., Braun, E., Borm, L. E., La Manno, G., et al. (2018). Molecular architecture of the mouse nervous system. *Cell*, 174(4):999–1014.

[Zheng et al., 2017] Zheng, G. X., Terry, J. M., Belgrader, P., Ryvkin, P., Bent, Z. W., Wilson, R., Ziraldo, S. B., Wheeler, T. D., McDermott, G. P., Zhu, J., et al. (2017). Massively parallel digital transcriptional profiling of single cells. *Nat Commun*, 8:14049.

[Zhu et al., 2017] Zhu, J.-Y., Park, T., Isola, P., and Efros, A. A. (2017). Unpaired image-to-image translation using cycle-consistent adversarial networks. In *IEEE Int Conf Comp Vision*, pages 2223–2232.
